# Artificial Neural Network Assisted Error Correction for MLC NAND Flash Memory

**DOI:** 10.3390/mi12080879

**Published:** 2021-07-27

**Authors:** Ruiquan He, Haihua Hu, Chunru Xiong, Guojun Han

**Affiliations:** 1 ZTE School of Information Technology, Xinyu University, Xinyu 338025, China; Ray_HRQ@outlook.com (R.H.); xcrmcu@163.com (C.X.); 2 School of Information Engineering, Guangdong University of Technology, Guangzhou 510006, China; haihua@mail2.gdut.edu.cn

**Keywords:** NAND flash memory, artificial neural network, error correction code, reliability

## Abstract

The multilevel per cell technology and continued scaling down process technology significantly improves the storage density of NAND flash memory but also brings about a challenge in that data reliability degrades due to the serious noise. To ensure the data reliability, many noise mitigation technologies have been proposed. However, they only mitigate one of the noises of the NAND flash memory channel. In this paper, we consider all the main noises and present a novel neural network-assisted error correction (ANNAEC) scheme to increase the reliability of multi-level cell (MLC) NAND flash memory. To avoid using retention time as an input parameter of the neural network, we propose a relative log-likelihood ratio (LLR) to estimate the actual LLR. Then, we transform the bit detection into a clustering problem and propose to employ a neural network to learn the error characteristics of the NAND flash memory channel. Therefore, the trained neural network has optimized performances of bit error detection. Simulation results show that our proposed scheme can significantly improve the performance of the bit error detection and increase the endurance of NAND flash memory.

## 1. Introduction

NAND flash memories have been widely used in smartphones, personal computers, data centers, etc. Thanks to these two key technologies: (1) continued scaling down process technology and (2) multilevel (e.g., MLC, TLC) cell data coding, the storage density of a NAND flash memory has been significantly increased over previous decades [1]. However, these two key technologies bring about a challenge in that the data stored in NAND flash memory may suffer from low reliability [2,3,4]. Furthermore, there are two major sources of noise in flash memory: cell-to-cell interference (CCI) and retention noise. Numerous works have been proposed to mitigate noises in NAND flash memory. For example, the data post compensation and predistortion technique [5] and detector design using a neighbor-a-priori information technique [6] exploit the a-priori information of the neighboring cells to mitigate the CCI. However, when considering retention noise, the voltage offset of flash memory cell tends to become unknown. It may be hard to use the a-priori information of the neighboring cells to compensate for the voltage shift caused by CCI. In addition, the CCI removal technique proposed by Lin [7] suffers from a similar problem in that the proposed technique ignores the impact of noise. In addition, Reference [8] proposed a retention-aware belief-propagation (BP) decoding scheme to mitigate the retention noise effect but did not take CCI into consideration.

Against the above background, the recent advances in neural networks and machine learning provide a new perspective to increase the reliability of MLC NAND flash memory. The key idea of the neural network is to learn an optimal network model from the massive training data, instead of using a definitive algorithm that is derived from a pre-defined model [9]. A pioneering work is reported in [10,11], which utilizes an artificial neural network to predict the threshold voltage distribution of NAND flash memory. In the pretesting, the above method assumes that the prior information of the retention time is informed in advance. When the flash controller is powered off, we cannot obtain the retention time.

In this paper, we use the neural network to learn an optimal network model to detect the bits errors in the cells that are disturbed by both CCI and retention noise and propose a neural network-assisted error correction scheme. However, it is difficult to record the retention time in a practical system, which means that accurate LLR values cannot be calculated. Therefore, we propose using relative LLR to estimate the actual LLR. The relative LLR is affected little by retention time, so we do not require retention time as an input parameter of the neural network.

In this paper, we first model the threshold voltage distribution as a Gaussian mixture model, which is fairly close to the voltage distribution of the practical NAND flash memory, and we calculate the LLR of the theoretical threshold distribution using a quantization scheme. Then, the corresponding LLR of the actual threshold distribution is mapped according to the relative position of the optimal reading reference voltage. It is found that this idea makes the relative LLR values remain relatively steady throughout retention time, which allows us to avoid using retention time as an input parameter of the neural network. Finally, using the relative LLR to estimate the actual LLR, we train the neural network and use the trained network to recovery the bits that may be wrongly detected in the soft-decision detection or hard-decision detection.

The rest of this paper is organized as follows. The flash channel model is presented in Section 2. Section 3 introduces our proposed ANNAEC scheme. Numerical simulation results are presented in Section 4. The conclusions are drawn in Section 5.

## 2. Channel Model

Without loss of generality, the proposed ANNAEC is performed over a model-based MLC NAND flash memory. Based on [5,8,12], we can model threshold voltage, $V_{th}$, by

(1) $$V_{th}=V+n_{RTN}+▵V_{CCI}-n_{retention},$$

where *V* denotes the desired voltage level, $n_{RTN}$ denotes random telegraph noise (RTN), $▵V_{CCI}$ denotes the shift caused by *CCI* noise, and $n_{retention}$ denotes retention noise.

### 2.1. The Voltage Distribution of Programmed and Erased Cell

The number of charges in the NAND flash memory cell can be altered in the program and erase operation. It is well known that before being programmed, a flash memory cell must be erased. In the erase operation, the charges in the memory cell are removed from the floating gate, and the threshold voltage of the erased cell will be set to the lowest voltage. The threshold voltage distribution of an erased cell follows a Gaussian distribution, which is given by

(2) $$p_{e}\left(x\right)=\frac{1}{\sigma_{e}\sqrt{2\pi }}e^{-\frac{{\left(x-\mu_{e}\right)}^{2}}{2\sigma_{e}^{2}}}=N\left(\mu_{e},\;\sigma_{e}^{2}\right),$$

where $\sigma_{e}$ and $\mu_{e}$ are the standard deviation and the mean of the threshold voltage of the erased cell, respectively.

According to [5,8], the threshold voltage of a programmed cell follows a Gaussian distribution shown below:

(3) $$p_{p}\left(x\right)=\frac{1}{\sigma_{p}\sqrt{2\pi }}e^{-\frac{{\left(x-\mu_{p}\right)}^{2}}{2\sigma_{p}^{2}}}=N\left(\mu_{p},\;\sigma_{p}^{2}\right),\;$$

where $\sigma_{p}$ and $\mu_{p}\in \left\{\mu_{p_{01}},\;\mu_{p_{00}},\;\mu_{p_{10}}\right\}$ are the standard deviation and the mean of the threshold voltage of a programmed cell.

### 2.2. RTN

The electron capture and emission at the floating gate near the interface generate RTN, which is greatly impacted by flash memory P/E cycles [13]. As P/E cycles increase, the tunnel oxide of the floating gate transistor is gradually damaged and generates charge trapping in the oxide and interface states. RTN leads to a random fluctuation of cell threshold voltage and widens the voltage distribution. Hence, RTN is modeled with a Gaussian-like distribution [8], given as

(4) $$p_{r}\left(x\right)=\frac{1}{\sigma_{r}\sqrt{2\pi }}e^{-\frac{x^{2}}{2\sigma_{r}^{2}}}=N\left(0,\;\sigma_{r}^{2}\right),\;$$

where $\sigma_{r}=0.00027\times {PE}^{0.62}$, denotes the noise standard deviation.

### 2.3. CCI

**Figure 1:**
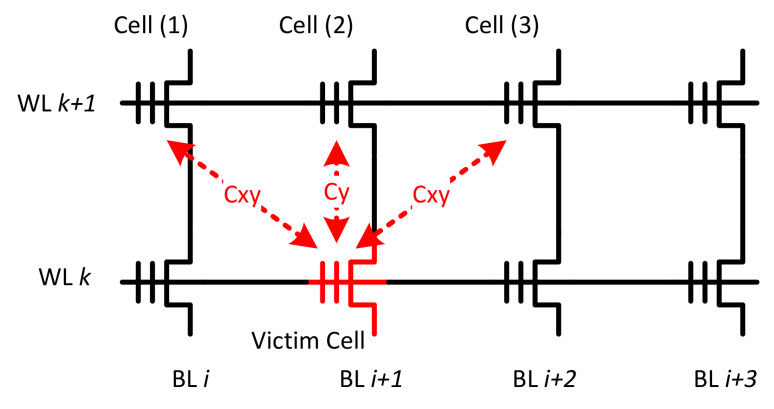
Illustration of the parasitic coupling capacitances among adjacent cells.

Because of the parasitic capacitance-coupling effect among adjacent cells in flash memory, the threshold voltage of the victim cell increases as the threshold voltage of an adjacent cell increases. The immediate adjacent cells are the major noise source of the CCI. We consider an all bit-line structure. As shown in Figure 1, when the (*k*+1)-th wordline (WL) has been programmed, the cell on the *k*-th WL can be programmed. Hence, the victim cell is influenced by three immediate adjacent cells. The threshold-voltage shift of the victim cell can be modeled as a linear combination of the threshold voltage changes of those immediate adjacent cells. We can estimate the threshold-voltage shift caused by CCI as

(5) $$▵V_{victim}=\sum_{n}\left(▵V_{t}^{\left(n\right)}\cdot \gamma^{\left(n\right)}\right),$$

where $▵V_{t}^{\left(n\right)}$ is the change of an immediate adjacent cell, which is programmed after the victim cell and $\gamma^{\left(n\right)}$ represents the coupling ratio. We assume the vertical and the diagonal coupling ratio are $\gamma_{y}$ and $\gamma_{xy}$, respectively. According to the cell-to-cell coupling strength factor *s*, we can set $\gamma_{y}=0.08s$ and $\gamma_{xy}=0.006s$ [12].

### 2.4. Retention

After a cell is programmed, the number of charges in the NAND flash memory cell continually reduce over time due to trap-assisted tunneling and charge detrapping [1]. Retention noise is modeled as a Gaussian distribution, i.e., $p_{t}\left(x\right)=N\left(\mu_{t},\sigma_{t}^{2}\right)=\frac{1}{\sqrt{2\pi }\sigma_{t}}e^{-\frac{{\left(x-\mu_{t}\right)}^{2}}{2\sigma_{t}^{2}}}$. The mean $\mu_{t}$, and the standard deviation $\sigma_{t}$, are given by

(6) $$\mu_{t}=▵V_{t}\left[A_{t}{\left(PE\right)}^{\alpha_{i}}+B_{t}{\left(PE\right)}^{\alpha_{o}}\right]\log\left(1+T\right),$$

(7) $$\sigma_{t}=0.3\left|\mu_{t}\right|,$$

where $▵V_{t}$ is the cell voltage change before and after being programmed, *T* donates memory retention time and PE is the number of PE cycles.

**Figure 2:**
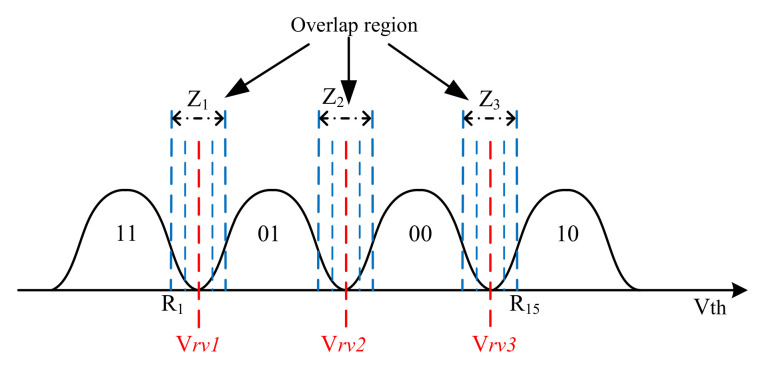
Illustration of 15-level uniform sensing quantization for multi-level cell (MLC) flash memory.

The conditional probability distribution function of the threshold voltage after being disturbed by RTN, CCI and retention are given as follows:

(8) $$\begin{array}{cc} p(V_{th}|k\in \left\{11,01,00,01\right\}) & =\frac{1}{64}[N(\mu_{k}-\mu_{t},\sigma_{k}^{2}\\ \; & +\sigma_{t}^{2}+\sigma_{r}^{2})+A+B+C], \end{array}$$

(9) $$\begin{array}{cc} A & =\sum_{\mu_{p}}[2N(\gamma_{xy}\left(\mu_{p}-\mu_{e}\right)+\mu_{k}-\mu_{t},\gamma_{xy}^{2}\left(\sigma_{p}^{2}+\sigma_{e}^{2}+2\sigma_{r}^{2}\right)\\ \; & +\sigma_{k}^{2}+\sigma_{t}^{2})+N(\gamma_{y}\left(\mu_{p}-\mu_{e}\right)+\mu_{k}-\mu_{t},\gamma_{y}^{2}(\sigma_{p}^{2}+\sigma_{e}^{2}\\ \; & \;\;\;\;\;\;\;\;\;\;\;\;\;\;\;\;\;\;\;\;\;\;\;\;\;\;\;\;\;\;\;\;\;\;\;\;\;\;\;\;\;\;\;\;\;+2\sigma_{r}^{2})+\sigma_{k}^{2}+\sigma_{t}^{2})], \end{array}$$

(10) $$\begin{array}{cc} B & =\sum_{\mu_{p}^{\left(1\right)}}\sum_{\mu_{p}^{\left(2\right)}}\sum_{\mu_{p}^{\left(3\right)}}N(\gamma_{xy}\left(\mu_{p}^{\left(1\right)}+\mu_{p}^{\left(2\right)}-2\mu_{e}\right)+\gamma_{y}\left(\mu_{p}^{\left(2\right)}-\mu_{e}\right)\\ \; & +\mu_{k}-\mu_{t},\left(2\gamma_{xy}^{2}+\gamma_{y}^{2}\right)\left(\sigma_{p}^{2}+\sigma_{e}^{2}+2\sigma_{r}^{2}\right)+\sigma_{k}^{2}+\sigma_{t}^{2}), \end{array}$$

(11) $$\begin{array}{cc} \; & C=\sum_{\mu_{p}^{\left(1\right)}}\sum_{\mu_{p}^{\left(2\right)}}N(\gamma_{xy}\left(\mu_{p}^{\left(1\right)}-\mu_{e}\right)+\gamma_{y}\left(\mu_{p}^{\left(2\right)}-\mu_{e}\right)+\mu_{k}\\ \; & \;\;\;\;\;\;\;\;\;\;\;\;\;\;\;\;-\mu_{t},\left(\gamma_{xy}^{2}+\gamma_{y}^{2}\right)\left(\sigma_{p}^{2}+\sigma_{e}^{2}+2\sigma_{r}^{2}+\sigma_{k}^{2}+\sigma_{t}^{2}\right))\\ \; & \;\;\;\;\;\;\;\;\;\;\;\;+\sum_{\mu_{p}^{\left(2\right)}}\sum_{\mu_{p}^{\left(3\right)}}N(\gamma_{xy}\left(\mu_{p}^{\left(3\right)}-\mu_{e}\right)+\gamma_{y}\left(\mu_{p}^{\left(2\right)}-\mu_{e}\right)+\mu_{k}\\ \; & \;\;\;\;\;\;\;\;\;\;\;\;\;\;\;\;\;\;\;-\mu_{t},\left(\gamma_{xy}^{2}+\gamma_{y}^{2}\right)\left(\sigma_{p}^{2}+\sigma_{e}^{2}+2\sigma_{r}^{2}\right)+\sigma_{k}^{2}+\sigma_{t}^{2})\\ \; & \;\;\;\;\;\;\;\;\;\;\;\;\;\;\;\;\;\;+\sum_{\mu_{p}^{\left(1\right)}}\sum_{\mu_{p}^{\left(3\right)}}N(\gamma_{xy}\left(\mu_{p}^{\left(1\right)}+\mu_{p}^{\left(2\right)}-2\mu_{e}\right)+\mu_{k}\\ \; & \;\;\;\;\;\;\;\;\;\;\;\;\;\;\;\;-\mu_{t},2\gamma_{xy}^{2}\left(\sigma_{p}^{2}+\sigma_{e}^{2}+2\sigma_{r}^{2}\right)+\sigma_{k}^{2}+\sigma_{t}^{2}), \end{array}$$

where $\mu_{p}^{\left(1\right)}$, $\mu_{p}^{\left(2\right)}$ and $\mu_{p}^{\left(3\right)}$ are the means of cells 1–3, respectively, which are shown in Figure 2, $\mu_{k}$ and $\sigma_{k}$ are the mean and standard deviation of the victim cell.

In this paper, we set the flash memory parameters as follows: $\mu_{p11}=1.2$, $\mu_{p01}=2.55$, $\mu_{p00}=3$, $\mu_{p10}=3.45$, $\sigma_{p}=0.05$, $\sigma_{e}=0.35$, $A_{t}=0.000035$, $B_{t}=0.000235$, $\alpha_{i}=0.62$ and $\alpha_{o}=0.30$.

## 3. Artificial Neural Network-Assisted Error Correction

In this section, we first present the idea of relative LLR calculation. Then we explain why an artificial neural network is useful for NAND flash memory. Finally, we introduce our proposed ANNAEC scheme.

### 3.1. Relative LLR

For soft decision belief-propagation (BP) decoding, a soft quantization scheme has been proposed. As an example, Figure 2 shows a 15-level uniform sensing quantization [12].

The overlap region is obtained by the entropy of the cell’s threshold voltage [12,14]. When the threshold voltage falls into the range $\left(R_{n-1},\;R_{n}\right]$, where $R_{n}$ is the *n*-th reference voltage, $R_{0}=-\infty$ and $R_{16}=+\infty$, the LLR values of the least significant bit (LSB) and the most significant bit (MSB) in the *i*-th cell can be calculated by (12) and (13), respectively:

(12) $$\begin{array}{cc} LLR_{lsb} & \left(R_{n-1},\;R_{n}\right)=\log\frac{\int_{R_{n-1}}^{R_{n}}p\left(V_{th}|11\right)+p\left(V_{th}|01\right)dx}{\int_{R_{n-1}}^{R_{n}}p\left(V_{th}|00\right)+p\left(V_{th}|10\right)dx}, \end{array}$$

(13) $$\begin{array}{cc} LLR_{msb} & \left(R_{n-1},\;R_{n}\right)=\log\frac{\int_{R_{n-1}}^{R_{n}}p\left(V_{th}|11\right)+p\left(V_{th}|10\right)dx}{\int_{R_{n-1}}^{R_{n}}p\left(V_{th}|01\right)+p\left(V_{th}|00\right)dx}. \end{array}$$

**Figure 3:**
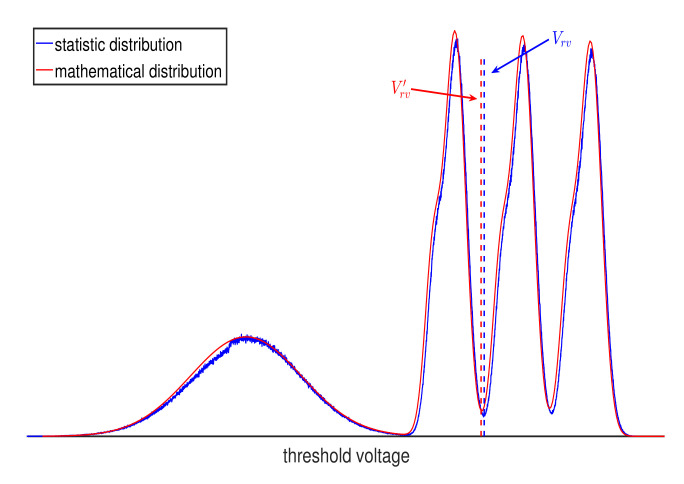
Illustration of the statistic distribution and mathematical distribution at s=1 and PE=1K.

However, it may be hard to accurately calculate the LLR values due to the retention noise. Even though retention noise is modeled as Gaussian distribution, the mean and the standard deviation are random, since $▵V_{t}$ is random as described in (6) and (7). Furthermore, it is difficult to obtain accurate retention time in a practical system. To deal with those problems, we can estimate LLR, based on the relative reference voltage positions, given as

(14) $$\begin{array}{cc} \; & LLR_{lsb}^{\prime }\left(R_{n-1}-V_{rv}+V_{rv}^{\prime },\;R_{n}-V_{rv}+V_{rv}^{\prime }\right)\\ \; & =\log\frac{\int_{R_{n-1}-V_{rv}+V_{rv}^{\prime }}^{R_{n}-V_{rv}+V_{rv}^{\prime }}p^{\prime }\left(V_{th}|11\right)+p^{\prime }\left(V_{th}|01\right)dx}{\int_{R_{n-1}-V_{rv}+V_{rv}^{\prime }}^{R_{n}-V_{rv}+V_{rv}^{\prime }}p^{\prime }\left(V_{th}|00\right)+p^{\prime }\left(V_{th}|10\right)dx}, \end{array}$$

(15) $$\begin{array}{cc} \; & LLR_{msb}^{\prime }\left(R_{n-1}-V_{rv}+V_{rv}^{\prime },\;R_{n}-V_{rv}+V_{rv}^{\prime }\right)\\ \; & =\log\frac{\int_{R_{n-1}-V_{rv}+V_{rv}^{\prime }}^{R_{n}-V_{rv}+V_{rv}^{\prime }}p^{\prime }\left(V_{th}|11\right)+p^{\prime }\left(V_{th}|10\right)dx}{\int_{R_{n-1}-V_{rv}+V_{rv}^{\prime }}^{R_{n}-V_{rv}+V_{rv}^{\prime }}p^{\prime }\left(V_{th}|01\right)+p^{\prime }\left(V_{th}|00\right)dx}, \end{array}$$

where $p^{\prime }$ means that we estimate $▵V_{t}$ in Equations (6) and (7) as $▵V_{t}\approx \mu_{k}-\mu_{e}$, $V_{rv}$ and $V_{rv}^{\prime }$ are the reference voltages of the actual threshold distribution and the theoretical threshold distribution, respectively, as shown in Figure 3, where $V_{rv}$ is obtained by voltage optimization [1] and $V_{rv}^{\prime }$ is obtained by theoretical calculations, such as minimizing entropy of the cell’s threshold voltage [12,14]. In (14) and (15), we first calculate the LLR of the theoretical threshold distribution using a quantization scheme. Then, the corresponding LLR of the actual threshold distribution is mapped according to the relative position of the optimal reference voltage.

**Figure 4:**
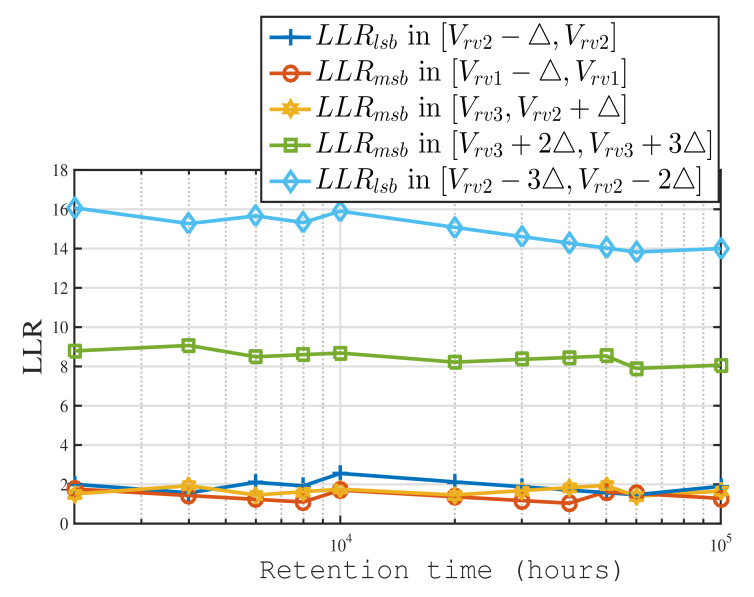
Plot of the relative log-likelihood ratio (LLR) versus data retention time at PE=1K, ▵=0.05 and s=1.

We depict the relative LLR versus data retention time in Figure 4. The relative LLR values remain relatively steady, which allows the neural network to not require retention time as an input parameter. In addition, LLR calculation is offline in a flash memory controller [15]. It may be difficult for a controller to estimate the characteristics of the memory channel because online estimation leads to a significant increase in the power consumption and read latency of the flash controller. Therefore, the proposed relative LLR can estimate the actual LLR over a time range, which can also help reduce the number of LLR tables stored in the controller.

### 3.2. Why Are Artificial Neural Networks Useful for NAND Flash Memory?

To simplify the analysis, this subsection first discusses the case that the CCI is only generated by the vertical neighboring cell. In this case, the conditional probability distribution function of the threshold voltage, (8), is simplified to (16):

(16) $$\begin{array}{cc} p & \left(V_{th}|k\in \left\{11,01,00,01\right\}\right)=\frac{1}{4}[N\left(\mu_{k}-\mu_{t},\sigma_{k}^{2}+\sigma_{t}^{2}+\sigma_{r}^{2}\right)\\ \; & \;\;\;\;+\sum_{\mu_{p}}N(\mu_{k}+\gamma_{y}\left(\mu_{p}-\mu_{e}\right)-\mu_{t},{\sigma_{k}}^{2}+\gamma_{y}^{2}(\sigma_{p}^{2}+\sigma_{e}^{2}\\ \; & \;\;\;\;\;\;\;\;\;\;\;\;\;\;\;\;\;\;\;\;\;\;\;\;\;\;\;\;\;\;\;\;\;\;\;\;\;\;\;\;\;\;\;\;+2\sigma_{r}^{2})+\sigma_{t}^{2}+\sigma_{r}^{2})]. \end{array}$$

**Figure 5:**
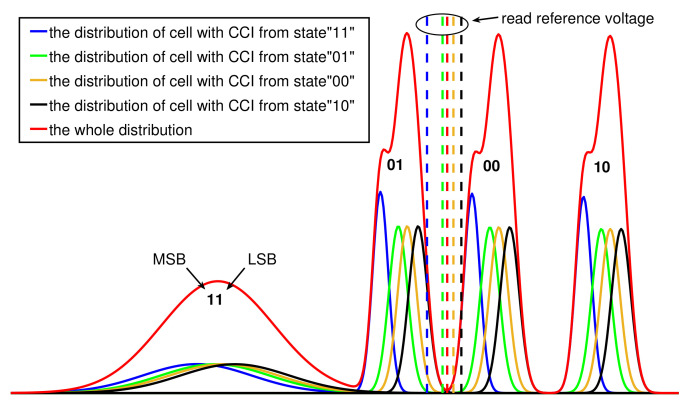
Illustration of the distribution of NAND flash memory at s=1.4 (the cell-to-cell coupling strength factor), PE=1K and $Retention\;time=10^{5}$.

In (16), it is seen that the threshold voltage distribution can be divided into four parts: the distribution of cells with CCI from “11”-state, “01”-state, “00”-state and “10”-state, which are also shown in Figure 4. In an overlap region, the bits with different CCI noise levels may have different error rates. For instance, in the overlap region between “01”-state and “00”-state, the bits of the cells in “00”-state with CCI from neighboring cells in “11”-state may be wrongly detected as “1” in LSB. In general, we want to find the optimal reading reference voltage at the intersecting point of the distributions of two states, such as the red dotted line in Figure 5. However, once we know the programmed state or the threshold voltage of the cells that donate the CCI to victim cells, the optimal reading reference voltage may change. For example, the optimal reading reference voltage should be selected by the blue dotted line in Figure 5, when the vertical neighboring cell is in the erased state.

**Figure 6:**
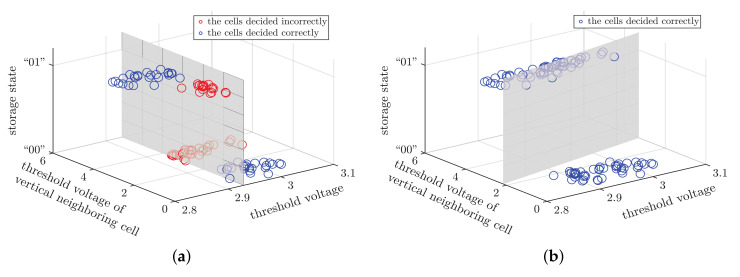
Illustration of the decision of least significant bit (LSB) in the NAND flash memory. (**a**) The conventional hard-decision plane in the three-dimensional coordinates. (**b**) The optimal plane.

In this paper, we expand the two-dimensional coordinates to three-dimensional, as shown in Figure 6a. The X-axis is the victim cell’s voltage, and the Y-axis is the threshold voltage of vertical neighboring cell. By doing so, one can easily find the incorrectly detected cells, marked with red dots. Moreover, we have two important observations:

(1) The correct cells (the blue dots) and the incorrect cells (the red dots) are not interlaced in the three-dimensional space. It means that the correct cells (or the incorrect cells) have similar features, which may be used for clustering them from the incorrect ones.
(2) The hard decision may not be the optimal decision when the surrounding cells have been read. In Figure 6a, the gray plane is the hard-decision plane, but not optimal. Suppose that there is a decision plane, shown as Figure 6b, and then we apply this plane to the same data in Figure 6a. One can see that the decision performance by the plane gets significantly improved compared to the plane in Figure 6a.

These two observations reveal that the detection of bits in a cell can be transformed into a clustering problem, which is to obtain an optimal classification hyperplane. When more surrounding cells are considered, the clustering problem will become more complex and the dimensions of the classification hyperplane will increase beyond three. To address this issue, We propose to use the neural network, which is good at solving various clustering problems.

### 3.3. Proposed Artificial Neural Network-Assisted Error Correction (ANNAEC) Scheme

**Figure 7:**
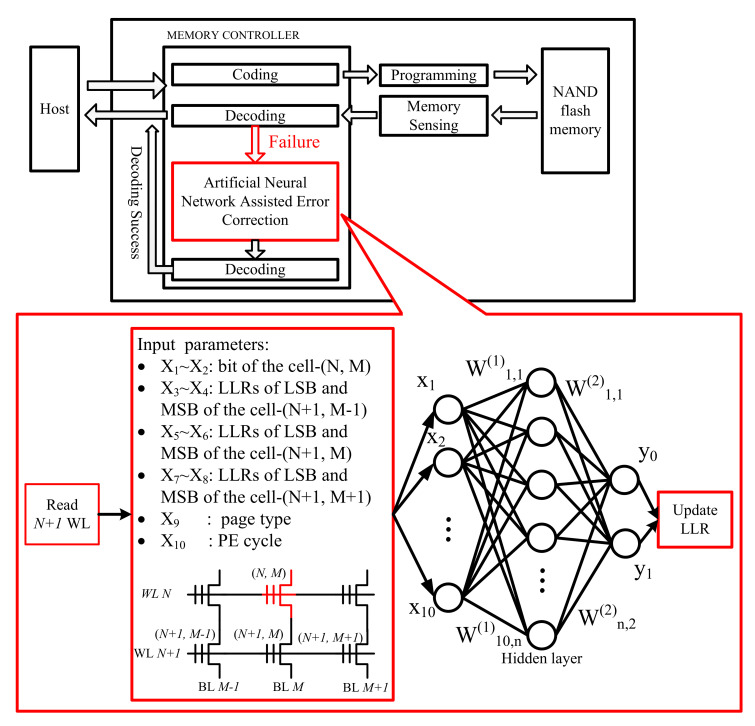
Block diagram of the proposed ANNAEC scheme in NAND flash memory.

The main idea of the proposed ANNAEC scheme is shown in Figure 7. In general, the flash memory controller uses soft-decision error correction [12], read-retry [1,16] and voltage optimization, which has been widely used in practical systems, to ensure the reliability of data stored in NAND flash memory. When these techniques are not effective in suppressing flash channel noise, the flash memory controller attempts to operate the proposed ANNAEC scheme to correct error bits. Moreover, it can reduce the power consumption and computation burden of the controller, since the cells in an overlap region take a relatively small part of the cells on a page.

In general, the host implements data writing and reading to the NAND flash memory chip by communicating with the memory controller, which communicates with the NAND flash memory chip. First, the host transfers data to the flash controller. The flash controller then encodes the data and writes it into the NAND flash memory chip. When the host reads the data, the flash controller communicates with the NAND flash chip. During this process, the NAND flash chip reads the data from the cell and sends it to the flash controller by reading the sensing circuit. After that, the flash controller corrects and restores the original data through the decoding algorithm and sends it to the host. The proposed a neural network assisted error correction algorithm is used as an alternative decoding algorithm. When the decoding of the flash controller fails, the neural network model is used to first correct the data and then perform decoding.

We label the positions of the cells in an overlap region, which is at the *N*-th word-line and the *M*-th bit-line in the block as (N,M), shown in Figure 7. The input parameters of the neural network are summarized in Table 1. $X_{1}$ and $X_{2}$ are the bits of cell-(N,M) in MLC memory, respectively. $X_{3}$∼$X_{8}$ are the LLRs of LSB and MSB of the immediate adjacent cells, i.e., cell-(N+1,M−1), cell-(N+1,M) and cell-(N+1,M+1). $X_{9}$ is the flag of page type. If the current reading page is LSB, we set $X_{9}$ to “0”; otherwise, $X_{9}$ is set to “1”. $X_{10}$ is the number of PE cycles. There are two reasons for choosing those parameters: (1) the threshold voltage is difficult to be obtained in a practical system, but the LLR and bits in a cell can help to locate the range of threshold voltage; (2) the vertical and the diagonal neighboring cells contribute about 81% of the CCI [17,18].

Afterward, we send the parameters into the back propagation neural network to correct error bits. The sigmoid function is selected as the activation function of the back propagation neural network, given as

(17) $$f\left(x\right)=\frac{1}{1+e^{-x}}.\;$$

The cost function is chosen as the typical mean square error (MSE) cost function [19], given by

(18) $$E=\frac{1}{2}\left[{\left(T_{y_{0}}-y_{0}\right)}^{2}+{\left(T_{y_{1}}-y_{1}\right)}^{2}\right],$$

where the outputs of neural networks $y_{0}$ and $y_{1}$ are the reliabilities of “0” and “1”, and *T* denotes the desired reliability in the data set. The relative LLR is calculated offline in the flash memory controller. It is difficult to recalculate the relative LLR, since the online characteristic estimation of the memory channel causes longer read latency. Since the accurate relative LLR is hard to recalculate, we update relative LLR by

(19) $$\begin{array}{cc} LLR_{update} & ={\left(-1\right)}^{\varepsilon +1}\left|LLR_{original}\right|, \end{array}$$

where $LLR_{original}$ denotes original relative LLR obtained in the sensing operation, and ε is given by

(20) $$\begin{array}{cc} \varepsilon & =\left\{\begin{array}{cc} 1 & \mathrm{if}\;y_{1}>y_{0}\\ 0 & \mathrm{else}. \end{array}\right. \end{array}$$

Although (19) does not update the accurate LLR to decode, it can estimate the value of LLR. Moreover, (19) is used to correct the sign of LLR, which is more important than the absolute value of LLR, since fewer error signs of LLRs fewer less error bits.

## 4. Experiment Results

### 4.1. Training

**Figure 8:**
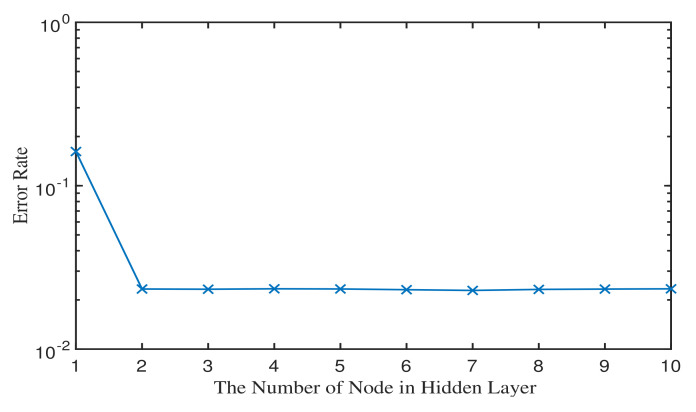
Performance of neural network under the different numbers of hidden layer nodes.

Throughout all experiments, we used a rate-0.9 (4544, 4096) QC-LDPC code and the BP decoding algorithm. The experimental platform is implemented in Matlab. The channel parameters, which are used to generate the training dataset, are shown in Table 2. Since the parasitic coupling capacitances of CCI are invariable in a flash memory ship, without loss of generality, we set the cell-to-cell coupling strength factor to be s=1. According to the raw bit error rate (RBER), we generate the dataset at PE={3000,4000,5000} and divide the dataset into two parts: error and correct bits, which are to be corrected, e.g., the cell-(N,M) in Figure 7. In total, the sizes of the training and validation data are 336,000 and 84,000, respectively. According to the performance of neural network versus the different numbers of hidden layer node, shown in Figure 8, the basic neural network structure is set to be {10,3,2}, meaning that there are 10 nodes in the input layer, 3 nodes in the hidden layer and 2 nodes in the output layer.

### 4.2. Performance

**Figure 9:**
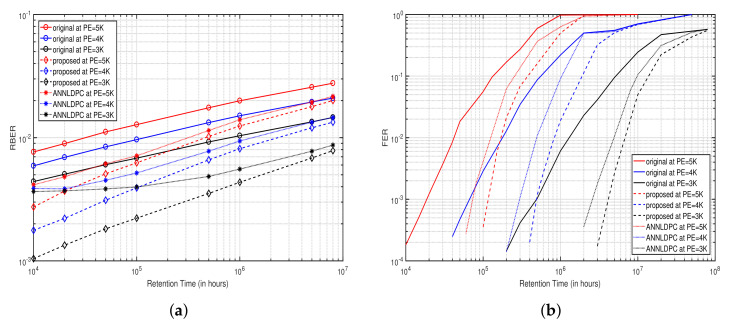
(**a**) Comparison of the raw bit error rate (RBER) performance of NAND flash memory with and without ANNAEC scheme versus data retention time at s=1. (**b**) Comparison of the frame error rate (FER) performance of low-density parity-check (LDPC) coded NAND flash memory with and without the ANNAEC scheme versus data retention time at s=1.

In Figure 9a,b, we compare RBER and frame error rate (FER) using ANN-LDPC [11], the proposed method and the original method without the neural network versus data retention time at s=1. We can observe that the proposed ANNAEC significantly reduces the RBER in comparison with the ANN-LDPC and original method.

For instance, in Figure 9a, the data retention time is about $3\times 10^{4}$ h at PE=5000 and RBER $=2\times 10^{-2}$, using the scheme without ANNAEC. Compared to the proposed ANNAEC scheme, Figure 9b shows that for the same performance, the ANN-LDPC can make the flash memory endure up to $3\times 10^{5}$ h and the proposed method provides a performance gain of approximately 67% of data retention, which makes the retention time of flash endure up to $5\times 10^{5}$ h. In addition, the proposed method has a more stable error correction performance, when the memory suffers from a weak interference. Similarly, we can notice that the proposed ANNAEC improves the FER performance by up to an error rate of $1\times 10^{-3}$ at a retention time of $4\times 10^{6}$ h and PE=3000. The ANN-LDPC has a FER performance of approximately $5\times 10^{-3}$.

## 5. Conclusions

In this paper, we have proposed to use the relative LLR calculation to estimate the actual LLR. Furthermore, in three-dimensional coordinates, we have transformed the bit detection problem into a clustering problem, which allows us to apply an artificial neural network in the memory channel. To solve the clustering problem, we proposed an artificial neural network-assisted error correction scheme, which has been shown by experiments to be effective in correcting the error bit when the conventional method without the neural network fails to decode. Simulation results have shown that the FER performance of our ANNAEC is significantly better than that of ANN-LDPC. For example, the ANN-LDPC can make the flash memory endure up to $3\times 10^{5}$ h, and the proposed method provides the performance gain of approximately 67% of data retention, which makes the retention time of flash endure up to $5\times 10^{5}$ h. Furthermore, our proposed approach can be extended to TLC or QLC flash memories.

## Figures and Tables

**Table 1:** Summary of input parameters.

Notation	Physical Meaning
$X_{1},X_{2}$	bit of the cell (N,M)
$X_{3},X_{4}$	LLRs of LSB and MSB of the cell-(*N* + 1, *M* − 1)
$X_{5},X_{6}$	LLRs of LSB and MSB of the cell-(*N* + 1, *M*)
$X_{7},X_{8}$	LLRs of LSB and MSB of the cell-(*N* + 1, *M* + 1)
$X_{9}$	page type (LSB:0; MSB:1)
$X_{10}$	PE cycle

**Table 2:** Training dataset (s=1).

	Retention Time (h)	*PE*	3000	4000	5000
RBER					
≈$6\times 10^{-3}$			$1\times 10^{5}$	$2\times 10^{4}$	$1\times 10^{4}$
≈$7\times 10^{-3}$			$2\times 10^{5}$	$4\times 10^{4}$	$1.5\times 10^{4}$
≈$8\times 10^{-3}$			$3\times 10^{5}$	$5\times 10^{4}$	$2\times 10^{4}$
≈$9\times 10^{-3}$			$5\times 10^{5}$	$1\times 10^{5}$	$3\times 10^{4}$
≈$1\times 10^{-2}$			$1\times 10^{6}$	$5\times 10^{5}$	$1\times 10^{5}$
Size of the training data			336,000		
Size of the validation data			84,000		

## Data Availability

The study did not report any data.

## References

[1] Cai Y., Ghose S., Haratsch E.F., Luo Y., Mutlu O. (2017). Error Characterization, Mitigation, and Recovery in Flash-Memory-Based Solid-State Drives. Proc. IEEE.

[2] Lee J.D., Choi J.H., Park D., Kim K. (2003). Data retention characteristics of sub-100 nm NAND flash memory cells. IEEE Electron Device Lett..

[3] Peng Z., He R., Han G., Cai G., Fang Y. (2019). Neighbor-A-Posteriori Information Assisted Cell-State Adaptive Detector for NAND Flash Memory. IEEE Commun. Lett..

[4] Xiong Q., Wu F., Lu Z., Zhu Y., Zhou Y., Chu Y., Xie C., Huang P. (2018). Characterizing 3D Floating Gate NAND Flash. ACM Trans. Storage.

[5] Dong G., Li S., Zhang T. (2010). Using Data Postcompensation and Predistortion to Tolerate Cell-to-Cell Interference in MLC nand Flash Memory. IEEE Trans. Circuits Syst. I Regul. Pap..

[6] Adnan Aslam C., Guan Y.L., Cai K. (2016). Detector for MLC NAND Flash Memory Using Neighbor A-Priori Information. IEEE Trans. Very Large Scale Integr. (VLSI) Syst..

[7] Lin X., Han G., Ouyang S., Li Y., Fang Y. (2018). Low-complexity detection and decoding scheme for LDPC-coded MLC NAND flash memory. China Commun..

[8] Aslam C.A., Guan Y.L., Cai K. (2018). Decision-Directed Retention-Failure Recovery With Channel Update for MLC NAND Flash Memory. IEEE Trans. Circuits Syst. I Regul. Pap..

[9] Riaz H., Park J., Choi H., Kim H., Kim J. (2020). Deep and Densely Connected Networks for Classification of Diabetic Retinopathy. Diagnostics.

[10] Wei D., Qiao L., Hao M., Feng H., Peng X. (2019). Reliability prediction model of NAND flash memory based on random forest algorithm. Microelectron. Reliab..

[11] Nakamura T., Deguchi Y., Takeuchi K. (2019). Adaptive Artificial Neural Network-Coupled LDPC ECC as Universal Solution for 3-D and 2-D, Charge-Trap and Floating-Gate NAND Flash Memories. IEEE J. Solid State Circuits.

[12] Dong G., Xie N., Zhang T. (2011). On the Use of Soft-Decision Error-Correction Codes in nand Flash Memory. IEEE Trans. Circuits Syst. I Regul. Pap..

[13] Compagnoni C.M., Ghidotti M., Lacaita A.L., Spinelli A.S., Visconti A. (2009). Random Telegraph Noise Effect on the Programmed Threshold-Voltage Distribution of Flash Memories. IEEE Electron Device Lett..

[14] Aslam C.A., Guan Y.L., Cai K. (2016). Read and Write Voltage Signal Optimization for Multi-Level-Cell (MLC) NAND Flash Memory. IEEE Trans. Commun..

[15] Sandell M., Ismail A. (2021). Machine learning for LLR estimation in flash memory with LDPC codes. IEEE Trans. Circuits Syst. II Express Briefs.

[16] Yong K.-K., Chang L.-P. (2020). Error Diluting: Exploiting 3-D NAND Flash Process Variation for Efficient Read on LDPC-Based SSDs. IEEE Trans. Comput. Aided Des. Integr. Circuits Syst..

[17] Kim T., Kong G., Weiya X., Choi S. (2013). Cell-to-Cell Interference Compensation Schemes Using Reduced Symbol Pattern of Interfering Cells for MLC NAND Flash Memory. IEEE Trans. Magn..

[18] Park S.K., Moon J. (2021). Characterization of Inter-Cell Interference in 3D NAND Flash Memory. IEEE Trans. Circuits Syst. I Regul. Pap..

[19] Žalik K.R. (2008). An efficient k^′^-means clustering algorithm. Pattern Recognit. Lett..

